# An analysis of common ethical justifications for compassionate use programs for experimental drugs

**DOI:** 10.1186/s12910-016-0145-x

**Published:** 2016-10-18

**Authors:** Kasper Raus

**Affiliations:** 1 Department of Philosophy and Moral Sciences, Ghent University, Ghent, Belgium; 2 End-of-Life Care Research Group Vrije Universiteit Brussel (VUB) & Ghent University, Blandijnberg 2, 9000 Ghent, Belgium

**Keywords:** Research ethics, Compassionate use, Expanded access, Analysis of common justifications

## Abstract

**Background:**

When a new intervention or drug is developed, this has to pass through various phases of clinical testing before it achieves market approval, which can take many years. This raises an issue for drugs which could benefit terminally ill patients. These patients might set their hopes on the experimental drug but are unable to wait since they are likely to pass away before the drug is available. As a means of nevertheless getting access to experimental drug, many seriously ill and terminally ill patients are therefore very willing to participate in randomised controlled trials. However, only very few terminally ill patients are able to actually participate, and those that do participate are at risk of participating *solely* as a way of getting experimental drugs. Currently, there are, however, ways of getting access to drugs that have not (yet) gained market approval. One such mean is via expanded access or compassionate use programs where terminally ill patients receive experimental new drugs that are not yet market approved. In this paper, I examine some of the common justifications for such programs.

**Main body:**

The most frequently voiced justifications for compassionate use or expanded access programs could be put in one of three categories. First, there are justifications of justice, where compassionate use programs could be seen as a just or fair way to distribute experimental new drugs to patients who are denied access to RCT’s through no fault of their own. Second, such programs could be justified by reference to the ethical principle of beneficence where it could be claimed that terminally ill patients stand to benefit greatly at very little risk (as they are already dying). Third, there are considerations of autonomy where, it is claimed, patients should be able to exercise their autonomy and have access to such drugs if that is there free choice and they are fully aware of the risks associated with that choice.

**Short conclusion:**

In this paper, I argue currently all justifications are potentially problematic. If they truly form the basis for justification, compassionate use programs should be designed to maximize justice, beneficence and autonomy.

## Background

Imagine you have a terminal illness. Your physicians informs you that in attempting to treat your illness, all known interventions or treatments have been tried, but have all shown to be ineffective. This effectively leaves you without viable treatment options. This situation is a reality for a significant amount of patients who then, ideally, receive palliative care to attempt to provide the best possible end of life. However, imagine your physician informs you that she is aware of a new drug that is currently in development. This drug has had promising results in laboratory and animal testing, and has also undergone phase I and phase IIa testing on human participants. Currently, a phase IIb randomised clinical trial (RCT) is being set up. Imagine you set your hopes on this new drug and would like to get access to it, what are your options?

One option is to wait for the drug to get market approval and reach the market. However, such testing takes time. Available research suggests that the average time from the first clinical tests on humans (phase 0 or I) to market approval is more than 7 years [1]. Terminally ill patients are thus likely to pass away before this medical intervention reaches the market, making this an unviable option.

Another alternative is to attempt to get enrolled in randomised controlled trial (RCT) for the drug which gives you a decent chance of receiving the experimental new agent. It is not assured you receive the drug since there is likely to be a control group receiving a placebo or the best currently available treatment and you could be (randomly) assigned to this group. Such clinical trials have a strong allure for terminally ill patients, and it is known that they will often ‘try anything to stay alive’ [2].

However, such a strong willingness of terminally ill patients potentially poses problems. First, it might make the offer or option to participate in an RCT a coercive one that some patients are unable to refuse. This might invalidate free and informed consent [3], although others have argued that this is not necessarily the case [4]. Second, it has been argued that it might even compromise the clinical trial system. Schuklenk (2014), for example, uses the example of AIDS trials in the past and argues that: ‘[Patients] lied and cheated to get into trials and left trials in such large numbers as to threaten the viability of the AIDS clinical trials system.’ ([5]: p.21).

As such, there is increasing attention for other ways to provide terminally ill patients with access to experimental and innovative drugs. As argued by Schuklenk:

*‘I doubt there exists currently a societal consensus anywhere that it is acceptable to compel catastrophically ill patients to participate in placebo controlled, randomized, double-blind trials as the only means of accessing investigational new agents’* ([5]: p.21).

One such mean is via expanded access programs or compassionate use programs.

### Compassionate use and expanded access programs^1^

In compassionate use or expanded access programs seriously ill patients without access to legally marketed drugs for their condition are granted access to experimental new drugs. This often concerns drugs (e.g. oncology drugs) in phase II or phase III of clinical trials.

Many legislations worldwide have such a recognized system of providing seriously ill patients with as-of-yet unapproved drugs, but regulations are known to differ significantly between legislations [6, 7]. However, what is common to most legislations is the condition that unapproved drugs should only be provided to seriously ill or dying patients and when there is some evidence of a possible effectiveness (e.g. from a phase I trial of the drug).

Such access to unapproved drugs for terminally ill patients outside of an RCT seems to enjoy considerable public support. There have, for example, been several highly mediatised cases of patients who were denied access to a clinical trial, but who nevertheless demanded access to the experimental drug tested in that trial. Two famous examples are those of Abigail Burroughs [8] and Josh Hardy [9]. There is also a growing phenomenon of patients using the internet and social media campaigns in an attempt to nudge pharmaceutical companies in order to gain access to experimental new drugs. Several online petitions for individual patients included more than 100,000 signatures [10]. Also, a website such as ‘Mytomorrows’ that sells access to experimental and unapproved drugs operates in 73 countries and currently has almost 4000 registered patients [11]. Partly because of the public support, a total of 24 US states have passed (or are in the process of passing) so called ‘Right-to-Try Laws’, that make explicit the right of seriously ill patients (under certain strict conditions) to receive interventions or drugs that have not yet received market approval [12, 13].

### Categorization of the arguments

However, despite the significant public support, compassionate use has been the focus of considerable debate. In this paper I try to shed light on the issue by bundling key justifications for compassionate use or expanded access. For each justification I attempt to see whether and when such justifications might be valid.

Looking at the justifications found in international literature, I believe them to fall within one of three broad categories. Some justifications apply to the ethical principle of justice, others refer to the principle of beneficence and, finally, some invoke the principle of autonomy. Each of these categories will be elaborated on below. Using these categories, I believe, provides a good perspective for reviewing the current debate on compassionate use.

My choice for the categories of justice, beneficence and autonomy is motivated by the fact that they are all widely recognized as ethical principles. Moreover they constitute broad categories and are not necessarily tied to a single ethical framework. These categories or principles are therefore able to serve as a heading to capture different specific arguments.

The use of a broad framework is also immediately relevant for this paper in which I will, in various places, compare compassionate use programs with randomised controlled trials (RCT’s). It may be argued that comparing both could be problematic as compassionate use is part of clinical practice and thus differs in relevant ways from the research context of RCT’s. This will not be expanded upon since discussing all the similarities and differences between research and clinical practice does not fall under the scope of this paper [14]. However, despite the possible differences, both clinical practice and research are (or should be) guided by these three ethical principles. Hence regardless of whether one sees research and clinical practice as requiring different ethical frameworks, comparison becomes possible by referring to shared principles.

## Main text

### First justification for compassionate use programs: justice and/or fairness

A first possible justification for compassionate use programs refers to the ethical principle of justice or fairness. Of course, many different approaches to justice exist (e.g. distributive, egalitarian, libertarian, etc.). However, as the label ‘justice’ is used here as a broad category to cover possible justifications for compassionate use, the concept will not be further specified here.

#### Consequences of the current system

As mentioned above, it may be an option for terminally ill patients who set their hopes on an innovative new drug to try to get enrolled in an RCT. One may become aware of such ongoing trials through health care professionals, but it is also possible for patients to search for clinical trials online via open clinical trials registries or websites such as ‘MyTomorrows’ [11].

However, despite the strong desire of some terminally ill patients to receive experimental drugs by participating in randomised controlled trials, only a few will be able to actually participate. First, phase I and II clinical trials often do not include many participants, so that only a selected number of terminally ill patients who want to participate can actually do so. Second, for a great deal of patients the clinical trial for a certain drug might not be conducted in their vicinity. Going long distances to participate in a trial might be an option for a determined patient, but is often difficult for a terminally ill patient who might be in poor physical condition.

Third, many seriously ill patients will not fit the inclusion criteria for a trial. In most clinical trials a significant amount of potential participants is excluded, in order to create a homogeneous group. Patients in poor physical condition are often excluded (as they might die before the completion of the study or are taking too many other drugs (creating confounding factors), etc.). Some research suggests that the rate of exclusion for phase I trials based on poor physical state can be 20 % [15] and even up to 35 % [16]. In general, those worst off (e.g. those with a rapidly progressing illness) are most likely to be denied participation. In other cases, a clinical trial might be aimed at a particular condition or medical problem, thereby excluding patients with other (similar) conditions. This was the case in the famous Abigail Burrough case where an American student diagnosed with head and neck cancer was unable to participate in a clinical trial, since the trial was restricted to patients with colon cancer. Her father, Frank Burroughs, considered this unfair and (unsuccessfully) sued the FDA.

It is also important to remark that not all patients who *do* participate in the clinical trial, actually receive the experimental drug, as most clinical trial consist of a control group that receives a placebo or best available treatment. Since we are here concerned with terminally ill patients, there is unlikely to be a best available treatment, so it will mostly concern a placebo control group.

Recently, a new issue with inclusion and exclusion arose when Uppsala University started a clinical trial for a drug for patients with neuroendocrine cancer. For this trial, an American millionaire diagnosed with neuroendocrine cancer provided considerable funding in exchange for participation in the trial. In a way, this patient bought his way into the trial, thereby also bypassing randomisation, as he would be allocated to the intervention group. Researchers who are part of this trial are now trying to explore whether it is possible to create a system where rich patients fund clinical trials in exchange for participation [17].

In short, whether patients who set their hopes on a certain innovative drug are able to participate in a certain trial is determined by various factors outside of the patient’s control: distance to the trial, the number of participants allowed in the trial, the condition on which the drug is tested, the patient’s physical condition. In the case of the Uppsala University trial it also dependent on one’s wealth. Of course, as correctly pointed out by a reviewer, that participation depends at least partly on factors beyond one’s control does not necessarily make it unfair. For example, trials have to be conducted somewhere and so will, by necessity, take place close to some patients and further away from others. In other cases exclusion of some patients might be grounded and justified to guarantee the scientific rigour and validity of a trial. However, regardless of the fairness, inherent to the strict methodology of RCT’s is that a great deal of patients will be denied access.

#### Compassionate use or expanded access programs

One might argue that compassionate use or expanded access programs could provide a fair way of distributing experimental drugs to patients who, through no fault of their own, are excluded from participating in trials. In compassionate use programs, access to the drug depends less on one’s geographical location, as drugs can be shipped and there is less monitoring involved. Via compassionate use programs, drugs could also be supplied to patients in poor physical condition who are now mostly excluded from participation.

However, as mentioned above, pharmaceutical companies are free to refuse providing drugs in the compassionate use program, and refusal currently does not have to be justified. If drug companies agree to a compassionate use program, they are therefore allowed to allocate the drugs in any way they see fit. The FDA in its communication with patients, for example, reports that:

*The [drug] company may not have enough of the drug available for all patients requesting expanded access. Some companies establish a lottery system to determine which patients will have treatment access. Others make the decision on a case-by-case basis* [18].

Therefore, according to most current legislation, a compassionate use program might result in more people obtaining the experimental drug, but there is currently no guarantee that the drug is allocated in a way that is consistent or fair. As mentioned earlier, some cases are heavily mediatised and some patients conduct successful social media campaigns that could result in getting a pharmaceutical company to grant their request [10]. In such cases, drugs are being distributed to those who are fortunate to have their case mediatised or whose online campaign is successful. It is also possible to purchase experimental drugs online, in which case the service is available to those who can afford them.

Some companies *are* considering ethical ways to handle compassionate use requests. Quite recently Janssen has created a Compassionate Use Advisory Committee (CompAC) which contains physicians, bioethicists, patients and patient representatives and advises on compassionate use requests [19]. This indicates that compassionate use programs *could* be designed to guarantee fairness in allocation and are thus not *inherently* unfair. I would argue that one can only justify compassionate use by reference to justice or fairness if the distribution of drugs is guaranteed to be fair or just. As pharmaceutical companies are now free to distribute compassionate use drug, it is highly questionable whether this condition is fulfilled. Forcing companies participating in compassionate use programs to use a well thought out and fair process of distribution would be a good idea, in my opinion.

#### Alternatives

Even if RCT’s are not the best way to do test drugs that might benefit terminally ill (because of the presence of a placebo control group or the limited number of participants), compassionate use programs do not constitute the only alternative. As mentioned earlier, designing and testing a drug is a lengthy process, but there might be ways to speed up the drug approval process. For example by using a Expansion Cohort Design where, instead of conducting different phase I, II and III studies, one adds cohorts to the initial first-in-human trial based on the incoming data [20, 21]. This potentially allows for more flexibility and takes up considerably less time. If the time-to-approval is significantly reduced, waiting for a drug to be approved may indeed become a viable option for more terminally ill patients.

Another might be to use ‘n-of-1’ trials. In such single patients trials, patients might receive experimental new drugs, but data is gathered in a more scientific way than in most current compassionate use programs [22]. In this way, we provide early access but we do not ‘abandon our commitment to well-designed, well-conducted clinical trials’ ([20]: 2003).

Alternative designs might, off course, also be possible [23]. Relevant here is the fact that even if one argues that RCT’s are ethically suboptimal when it concerns terminally ill patients, several alternatives to compassionate use programs are also viable and might even pose considerable advantages.

### Second justification for compassionate use programs: beneficence

The justification for compassionate use programs seems to be that providing seriously ill patients with experimental drugs provides them with benefits and could save or prolong their lives.

However, this assumption could be considered questionable. There seem to be two types of risk. First, there is a direct risk for the intervention or pharmaceutical compound one takes to have harmful effects on patients. Second, there is a risk that by participation in the compassionate use programs patients are exploited for the benefit of researchers or pharmaceutical companies.

#### Risks of harmful side-effects

The ingestion of experimental drugs is fraught with risks and it is known that only a very small percentage of drugs that are clinically tested on humans actually end up being marketed [24]. Therefore, it is highly possible that the drug being given is actually harmful or ineffective. For example, research suggests that of all oncology drugs that enter phase I, only about 26 % ends up getting approved, for oncology drugs entering phase II and II this is, respectively, 34 and 57 % [25]. Even for drugs that make it to phase III, a significant amount never make it to the market. It is highly uncertain whether governments or health insurance companies are willing to cover health costs resulting from unapproved drugs. As such seriously ill patients taking such drugs run significant health and financial risks.

Moreover, risks are not limited to a directly harmful effect of the investigational drug or intervention, as there might, for example, also be other financial harm. Some drugs might be ineffective. If, as is the case in some countries, patients in compassionate use programs pay pharmaceutical companies for these drugs, terminally ill patients, who are often already financially burdened, risk additional financial burdens for a drug that does not work properly or does not work at all.

Of course, even taking market approved drugs is always potentially risky. Drugs that are approved for market have been successfully tested in RCT’s, but most patients in clinical practice do not fulfil the RCT inclusion criteria. One should therefore be careful not to overstate benefits for every individual patient of an approved drug. Even approved drugs do not work (properly) in a significant number of patients [26] or might result in severe adverse events. Therefore, stating that unapproved drugs are always dangerous whereas approved drugs are safe and effective is an oversimplification and wrong on an individual patient level. Nevertheless, although obtaining market approval is not a *perfect* guarantee for safety and effectivity, it at least provides some guarantees of being safe and effective on a population level.

Naturally, harmful side effect can also occur in clinical trials. However, such trials are small scale and closely monitored. This differs from compassionate use programs where, currently there is often no duty to report side-effects [6]. Also, should any dramatic side-effects occur during a clinical trial, this is limited to participants in that clinical trial, all of whom are (or should be) under close medical observation and clinical trials can also be stopped when necessary.

It is also important to note that there is a significant difference between compassionate use programs (which are clinical practice) and RCT’s (which are research). In RCT’s individual participant risks are balanced not only against individual benefits, but also against the scientific and social value of the results of the research study for other patients in similar situations. This could justify some individual risks. In compassionate use programs (being clinical practice) one has an obligation solely towards the individual patient, making harm or risks of harm potentially less acceptable.

#### Risk of exploitation

In compassionate use programs, one should be attentive to the potential of exploitation [27, 28], as there seems to be a risk that seriously ill patients could be used for financial gain or as cheap and easy research subjects.

First, it varies considerably from legislation to legislation whether pharmaceutical companies providing experimental drugs via compassionate use programs can charge patients. When drugs have to be provided free of charge, it is claimed, drug companies are unlikely to participate in a compassionate use program. Either they do not have a large supply of an experimental new drug, or they are afraid of bad publicity should the drug prove unsafe or ineffective [29, 30]. Allowing companies to charge money then functions to overcome that reluctance, as for example argued by Darrow (2015):

*For example, a manufacturer’s reluctance to provide product because of financial concerns could be addressed by permitting companies to charge amounts closer to the likely postapproval cost of drugs* ([31]: p.285).

An alternative approach that has been suggested, is to put all profit made by pharmaceutical companies participating in compassionate use programs on an interest-bearing account until approval is granted [32]. If the drug does not end up getting approved, the money on the account can be redistributed to health related projects. First, however, knowing what the profit is, requires pharmaceutical companies to report on the actual cost of developing and producing the drug, which is highly unlikely to happen. Second, in this scenario patients would still be charged money for a drug that is in no way guaranteed to be safe or effective. Third, for those drugs that eventually get approved, pharmaceutical companies could start making money before market approval is even granted.

In various other countries, such as Belgium, drugs provided via compassionate use programs should always be free of charge. This could avoid the danger of patients being exploited. However, such a risk still exist as, for example, websites do offer access to experimental drugs at a charge. This legal requirement is avoided by not selling directly to the patient.

Second, there is a risk that in compassionate use programs (terminally ill) patients are used as easy research participants. Compassionate use falls under clinical practice, but arguments have been made that those patients participating in a compassionate use program have a limited obligation to share some essential data (e.g. regarding the effect and any adverse events) [33]. In fact, in an increasing amount of cases, the results of compassionate use programs are reported as studies. However, as compassionate use programs are often not regulated the same way as clinical trials are, they might be less strict, which is a risk for patient safety. It might also be less costly as instead of paying participants (which often happens in clinical trials), participants of a compassionate use program do not receive any money and, in those countries where this is allowed, may even *pay* pharmaceutical companies for the drug. There is thus a fear that pharmaceutical companies may regard compassionate use programs as an easy way to do research, thereby potentially putting patients at risk:

*Blurring the lines between expanded access and clinical trials risks that expanded access programmes are undertaken as an easy way to collect information on new medicinal products instead of conducting randomised clinical trials. When this happens, the safeguards inherent to clinical trials, eg, having a control group, securing insurance to protect the patients, reporting all adverse events, reporting the results, etc. are all circumvented* ([6]: p.7-8).

This might in turn also blur the line between clinical practice and research. If one frames such a program as clinical practice, but uses it as a way to gather generalizable scientific data this constitutes research and it should follow the ethical standards for research.

If one indeed justifies compassionate use programs be reference to the principle of beneficence, they should be designed to provide maximum benefit and minimal risks. If pharmaceutical companies are allowed to charge patients money or use them as research participants for the company’s benefit, there is a significant risk of exploitation. Hence I would argue that if the principle of beneficence indeed justifies compassionate use programs, it justifies programs where drugs are provided free of charge and the program is set up as clinical practice focussing on the individual patient.

#### The case for palliative and/or dying patients

Above it has been questioned whether, overall, compassionate use is for the benefit of the patient, considering the risks associated with it. One could nonetheless maintain that palliative and/or dying patients constitute an exception. These patients are sure to pass away if they are not given the experimental drug so it could be argued that even though the chance of a benefit is small, so are the risks for palliative and/or dying patients. Walker et al. (2014), for example, argue that the most common argument for denying patients unproven medication relies on a precautionary principle that we are unaware of potential harms of experimental new drugs. They remark, however, that ‘this principle loses at least some of its moral force in cases where patients are otherwise at high risk of death or serious disability’ ([33]: p.5).

However, I believe this underestimates the possible harms palliative and/or dying patients could suffer. First, such patients are still at risk of being exploited, regardless of their prognosis. Second, even palliative and/or dying patients can be directly harmed, for example by spending their last days in unnecessary pain and agony [34, 35]. Finally, as argued above, there is also a financial risk. Compassionate use programs might cause already financially burdened patients to spend money on drugs that do not work. Some commentators have therefore argued that the fact that terminally ill patients face certain death does not do away with the ethical obligation to maximize benefit and minimize harm [36]. In compassionate use the physician’s ethical obligation does not differ from standard clinical practice where treatment is also associated with risks. In some cases where the risk of harm is disproportional to the potential benefit (as might be the case with drugs that are still being tested), this might, in my view, mean refusing to provide the patient with such an experimental drug.

### Third justification for compassionate use programs: autonomy

It may also be argued that providing terminally ill patients with another option to receive an experimental drug (besides RCT’s) could promote autonomy and make those RCT’s more ethical. Kodish (1991), for example, argued that clinical trials are *only* ethically justified if the intervention or drug in the clinical trial is also available outside of the RCT [3]. If an intervention or treatment is available both within and outside of a clinical trial, participants can freely chose to participate in either the RCT or the compassionate use program. Schuklenk therefore argues that not providing experimental drugs via compassionate use programs may amount to ‘coercing dying people into participating in particular trial designs’ ([37]: p.2). As such, compassionate use programs could result in a net gain in autonomy as it provides patients with two options where they previously had only one coercive option.

Two remarks might be made in response to this argument. The first is that there is a difference between a tempting offer and a coercive one. As already argued above, there is no doubt that many terminally ill patients are willing to try anything and that an offer to participate in an RCT is tempting. A study by Agrawal et al. (2006) into patients’ decision making regarding enrolling in phase I clinical trials found that: ‘More than 90 % of patients said they would still participate in the study even if the experimental drug caused serious adverse effects, including a 10 % chance of dying’ ([38]: p.4479). This suggests that many terminally ill patients are indeed willing to undergo great risks for a small glimmer of hope. For terminally ill patients, the background condition that they will inevitable pass away might make the offer coercive, but this is not *necessarily* the case. What makes offers coercive has been the topic of great debate [39], and whether or not an offer to participate in an RCT is coercive is not self-evident.

A second response is that the argument that compassionate use programs raise autonomy is only valid if and when patients do indeed get access to innovative drugs. As was already mentioned above, drug companies are always free to refuse to provide experimental drugs. Mere FDA or EMA approval and a hypothetical access to experimental drugs or interventions could hardly be said to raise autonomy for patients.

Regardless of whether or not autonomy is increased with compassionate use programs, the principle remains of great importance in justifying the practice. A commonly voiced argument in favour of compassionate use programs or early access to experimental drugs is that despite the potential risks, terminally ill patients should be able to freely choose to undergo such risks for the hope of improving their condition or lengthening survival [31]. Terminally ill patients are deemed capable to consent to participate in randomised clinical trials, including trials to test experimental drugs [40]. It could therefore be argued that they should also be deemed capable to consent to receiving experimental drugs (with all the risks associated with that consent). Automatically denying terminally ill patients the capacity for consent based on their medical condition seems paternalistic and unfounded.

However, there seem to be several red flags regarding autonomy or informed consent in the case of compassionate use programs. The first is that autonomy in terminally ill patients might be threatened by therapeutic optimism, especially in compassionate use programs. A second red flag or concern is that, unlike in an RCT, in a compassionate use program there is no central organ monitoring or overseeing consent.

First of all, compassionate use programs avoid the risk of therapeutic *misconception*, as, unlike in a clinical trial, the main goal of such a program is indeed therapeutic. There is, however, a serious risk of therapeutic *optimism*. Especially since a compassionate use program is not framed as ‘research’, thereby potentially causing participants to overlook or fail to understand the experimental nature of the drug. Much research has been done into hope, therapeutic misconception and therapeutic optimism [41, 42]. Generally most commentators seem to agree that while therapeutic misconception and optimism pose challenges for informed consent, they do not automatically invalidate informed consent [40]. It, however, remains an issue to monitor.

Second, in a compassionate use program there is often no central organ monitoring the consent process which runs via individual physicians. In Australia, for example, compassionate use programs are not seen as research and rarely go through a human research ethics committee. There is then no committee which oversees or checks the consent procedure. In fact, it has been suggested that this is deliberate and that ‘data collection is often kept to a minimum in order to avoid the need for such review’ [43]. Therefore, there is no real control over whether or not patients under the compassionate use program freely consent and truly understand the risks associated with such participation [44].

Hence, for the principle of autonomy to provide an adequate justification, measures should be taken to guarantee autonomy for patients. This means monitoring the quality and validity of informed consents given and being attentive to the possibility of excessive therapeutic optimism.

## Conclusions

In this paper the arguments given in favour of compassionate use or expanded access programs have been put into three broad categories. Compassionate use programs could be justified by claiming that they allow for a fair distribution of experimental drugs to patients with no other means to access the drug. Such compassionate use programs could also be justified by appealing to the ethical principle of beneficence. Terminally ill patients, one could argue, stand to benefit greatly and risk relatively little (as they are already facing inevitable death). In this case even a small benefit (e.g. a small chance of recovery or extended life-expectancy) outweighs the (small or non-existent) risks. Last, for the justification of compassionate use or expanded access programs appeals could be made to autonomy. Terminally ill patients are autonomous and are able to give free and informed consent. If terminally ill patients, the argument goes, freely take on the risks of an experimental drug for the chance of great benefit, who are we to refuse them?

However, I have argued that – currently – appeals to justice and beneficence are highly problematic. Pharmaceutical companies are, according to most current legislations, free to distribute drugs in compassionate use programs as they see fit. There is thus no guarantee that compassionate use or expanded access programs distribute drugs in a fair or equal way. Appeals to beneficence could, it was argued, be equally problematic. The assumption that terminally ill patients stand to lose little to nothing is questionable as they might be directly harmed, either physically (e.g. by spending their last days in agony) or financially (e.g. by paying a significant amount of money for an ineffective or unsafe drug). Such patients might also be harmed by being exploited as a source of money or as a source of easy research data.

Another way to justify compassionate use is by reference to the principle of autonomy. Terminally ill patients are effectively left without little viable options and refusing them to freely and autonomously consent to receiving an experimental drug could even deprive them of their last option. Patients, it could be argued, might be willing to take an experimental drug in full awareness of the risks associated with undergoing such a drug. Nevertheless, the possibility of therapeutic optimism does pose a risk for informed consent. This is combined with the fact that in compassionate use of expanded access programs there is generally no central organ monitoring the process and quality of informed consent. This means that although autonomy could provide justification, in practice there is little guarantee that terminally ill patients do indeed make fully autonomous decisions.

As such, I argue that if compassionate use is to be justified by reference to justice, beneficence and autonomy, measures must be taken to justify this reference. Companies participating in compassionate use programs should perhaps be required to distribute the experimental drugs fairly. Measures should also be taken to maximize benefits for patients and minimize risks. This could mean providing experimental drugs free of charge and setting up the compassionate use program as clinical practice and not as research. Finally for the principle of autonomy to justify compassionate use, measures should be taken to guarantee the validity and non-coercive nature of the informed consent given by those receiving experimental drugs. Currently, however, I believe compassionate use programs are not as just, not as beneficent and not as autonomous as they could and should be.

## Abbreviations

RCT: Randomised Controlled Trial

## Footnotes

1 ‘Both terms are synonyms used by different instances. For the remainder of this paper, I will adopt the term ‘compassionate use’.
